# Robotic Cholecystectomy Remains Safe and Effective After Regular Staffing Hours

**DOI:** 10.7759/cureus.54413

**Published:** 2024-02-18

**Authors:** Emanuel Shapera, Melissa Touadi, Jade Dickow, Ellie Azure, Melania Attar, Melinda Gorges, Marudeen Aivaz

**Affiliations:** 1 General Surgery, Scripps Clinic, San Diego, USA; 2 School of Medicine, University of South Florida, Tampa, USA; 3 Surgery, Academy of Our Lady of Peace, San Diego, USA; 4 Surgery, University of California San Diego, San Diego, USA; 5 Surgery, Zucker School of Medicine, Hempstead, USA

**Keywords:** robotic-assisted cholecystectomy, nursing shortage, staffing, access to health care, after-hours care

## Abstract

Background

Robotic-assisted surgery continues to grow in popularity. Access during evenings and weekends for non-elective operations can be restricted out of safety concerns. We sought to analyze and compare outcomes of patients undergoing robotic cholecystectomy, a common urgent procedure for acute calculous cholecystitis, during regular hours versus evenings or weekends. Based on this comparison, we sought to determine if this restriction is justified.

Methods

We performed a retrospective analysis of 46 patients who underwent robotic cholecystectomy for acute calculous cholecystitis per 2018 Tokyo criteria by a single surgeon at a single institution between 2021 and 2022. Patients were grouped as undergoing “after-hours” cholecystectomy if the operation started at five pm or later, or anytime during the weekend (Saturday, Sunday). Demographic, perioperative, and outcome variables were tabulated and analyzed. For illustrative purposes, the data presented as median ± standard deviation were applicable.

Results

After-hours cholecystectomy occurred in 26 patients and regular-hours cholecystectomy occurred in 20 patients. There were no significant differences in perioperative variables between the two cohorts in terms of body mass index, age, gender, cirrhotic status, American Society of Anesthesiology score, white blood cell count, or neutrophil percentage. The after-hours group had more prior abdominal operations. There were no significant differences between the two groups in terms of operative time, estimated blood loss, or length of stay. There were no mortalities. There was one readmission in the after-hours cohort unrelated to the operation.

Conclusion

Robotic cholecystectomy can be safely performed on the weekends and evenings. Hospitals should make the robotic platform available during this time.

## Introduction

Robotic surgery has provided an alternative to laparoscopy and open techniques to achieve the benefits of a minimally invasive operation with superior dexterity and three-dimensional optics. However, accessibility remains an important barrier to adoption among surgeons. Staff must be trained to operate the platform, which for multiple reasons can be difficult to obtain, particularly in the evenings and weekends when hospitals struggle to provide adequate nurses and technicians [1]. Many hospitals will limit the utilization of the robotic platform at the administrative level during these hours for non-elective operations, despite growing literature demonstrating the salutatory benefits of the robotic platform in intestinal and hepatobiliary cases [2-4]. With rising levels of burnout, problems in staff retention, and the effects of the coronavirus disease 2019 (COVID-19) pandemic, these concerns have become accentuated and must be addressed [5].

We sought to publish the outcomes of patients presenting with acute cholecystitis, one of the most common diseases treated by general surgeons, who underwent non-elective robotic cholecystectomy during the weekends and evenings and compare that to outcomes of patients undergoing the same operation during regular hours. We hypothesized that despite the aforementioned concerns, utilization of the robotic platform during these times is not dangerous and provides satisfactory and safe outcomes. We hope this data will convince hospitals across the country, often making decisions based on profit rather than facilitation of health, the importance of maintaining round-the-clock robotic surgical capabilities. The aim of our paper, therefore, is to demonstrate that robotic cholecystectomy is safe and feasible during hours when sub-optimal operating room staffing may occur, with the weekends and evenings being a surrogate for such hours.

This article was previously presented as a meeting podium presentation at the 2023 SLS Society of Laparoscopic Surgeons Annual Scientific Meeting on October 5, 2023.

## Materials and methods

Data source and exclusion criteria

Between 2021 and 2022, 46 patients who underwent robotic cholecystectomy by a single general surgeon at a single institution were included in the final retrospective analysis. The year 2021-2022 was chosen as this was appreciated to be the tail end of the COVID-19 pandemic; it was an intersection between two important events in recent healthcare history: the significant increase in non-emergency non-elective operations as lockdowns began to ease and the ongoing crisis in healthcare worker retention [6]. The surgeon in the study was hepatobiliary trained and well along the robotic learning curve at the beginning of the study year.

If the operation started at or after five pm, or on a weekend day (Saturday or Sunday) the case was placed in the “after-hours” group. If not, it was placed in the “regular hours” group. Inclusion criteria were thus: all patients who underwent non-elective robotic cholecystectomy for acute calculous cholecystitis per the 2018 Tokyo Criteria. If a patient underwent a concomitant procedure, they were excluded, since such an event could impact the perioperative course. The operating surgeon does not have criteria for choosing which patient undergoes laparoscopic or robotic cholecystectomy but utilizes the platform as its availability, the needs of the patient, and the schedule permits. A history of prior abdominal operations did not constitute a major contraindication to the robotic platform, and it has recently been shown to be safe to proceed with a robotic approach in these situations [7]. An Institutional Review Board waiver was granted due to the retrospective nature of the study.

Patient variables

The following clinical variables were noted: age, sex, body mass index (BMI), American Society of Anesthesiology (ASA) score, history of prior abdominal procedures, cirrhosis, estimated blood loss (EBL), operative time, intravenous fluids (IVF) given, length of stay (LOS), pathologic result, complications, 30-day readmission, and 30-day mortality. Complications were graded by the Clavien-Dindo Classification system (I-V). The following pre-surgical laboratory variables were noted: glucose level, white blood cell (WBC), neutrophil percentage, total bilirubin (Tbil), alkaline phosphatase (AP), aspartate transaminase (AST), alanine transaminase (ALT), and creatinine (Cre). Operative time was recorded from the start of the initial incision to the placement of the final dressing.

Operative technique

**Figure 1:**
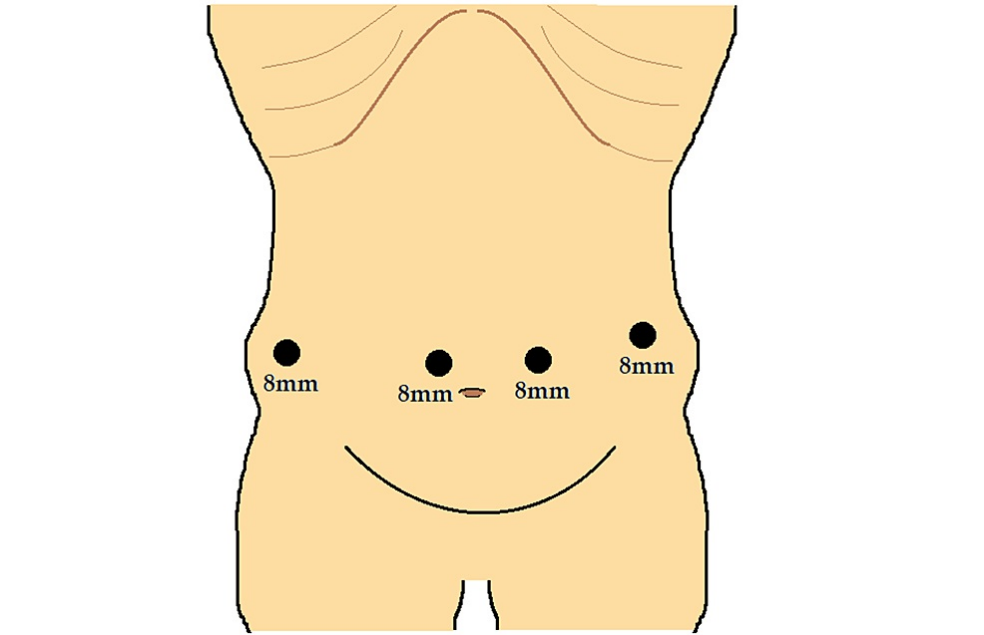
Port Placement for Robotic Cholecystectomy

The following is the surgeon’s technique for performing robotic cholecystectomy. Patients are positioned supine on the operating table and induced with general endotracheal anesthesia. The da Vinci Xi® robotic surgical system is docked and paired with the operating table to allow for intraoperative bed motion. An 8 mm trocar is inserted through the umbilicus for the robotic camera. Three 8 mm robotic ports are placed under direct laparoscopic visualization: one at the right anterior-axillary line and two at the left mid-clavicular and left anterior axillary lines (Figure 1).

**Figure 2:**
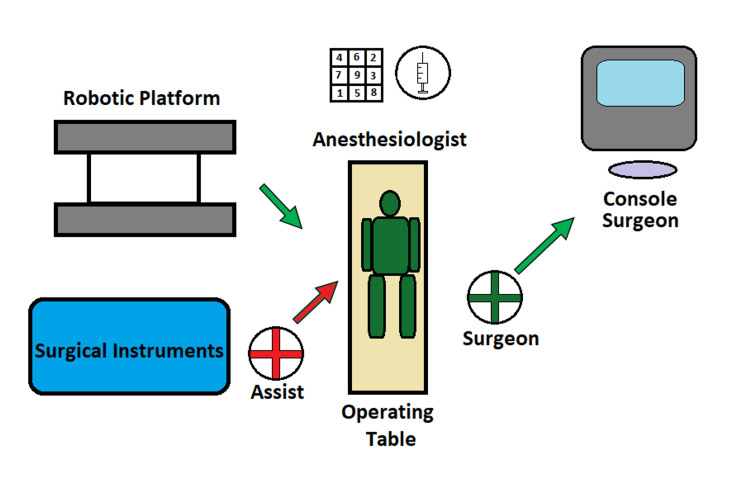
Operating Room Setup for Robotic Cholecystectomy

The following instruments are utilized from the patient’s right to left in increasing robotic arm numerical order: fenestrated bipolar, 30-degree camera, monopolar hook, and small grasper. The robotic system is docked over the patient's right shoulder. The bedside assistant is the scrub technician who performs instrument changes as needed (Figure 2).

The operation begins with adequate liver inspection by taking down the falciform ligament all the way up to the hepatocaval confluence. This permits the small grasper to perform cephalad retraction of the liver by grasping the falciform ligament if the latter is nodular and susceptible to cracking. As a vascularized pedicle, the mobilized falciform is also placed in the gallbladder fossa after resection to prevent a postoperative fluid collection or hematoma. The peritoneum is scored close to the infundibulum with the monopolar hook and carefully taken down. Right-angle action with the hook monopolar permits skeletonization of structures. Varicose veins encountered are destroyed with bipolar energy. Clipping of the cystic duct and artery occurs within the critical view of safety followed by division with scissors. Indocyanine green administered in the preoperative bay permits intraoperative fluorescent cholangiogram, routinely utilized. The gallbladder is then removed from the liver bed with monopolar energy and placed in a 12mm bag for retrieval via the umbilicus. The latter is then reconstructed with a slowly absorbable 0 monofilament suture in a figure-of-eight fashion at the fascia followed by a 4-0 absorbable monofilament suture of all skin incisions.

Patients are administered a regular diet upon leaving the post-anesthesia care unit and discharged home the next day pending normal labs and clinical parameters. 

Statistical analysis

Data was collected on an Excel (Microsoft Corporation, Redmond, Washington, United States) spreadsheet maintained on an encrypted portable hard drive device kept secured within a locked office. Data was analyzed using GraphPad InStat version (3.0) (GraphPad Software La Jolla, CA) and MedCalc Statistical Software version 20.218 (MedCalc Software Ltd, Ostend, Belgium; https://www.medcalc.org; 2023). For illustrative purposes, numerical data are presented as median ± standard deviation. The T-test was used to compare continuous variables (such as EBL, etc.) between the two groups and chi-square to compare categorical variables (gender, etc.).

## Results

There were 46 patients who met the inclusion criteria. After-hours cholecystectomy occurred in 26 patients and regular-hours cholecystectomy occurred in 20 patients. There were no statistically significant differences between the two groups with regard to age (60.6 ± 18.1 vs 63.9 ± 15.1 years old, p = 0.144), female sex (65% vs 80%, p = 0.275), BMI (31.1 ± 6.38 vs 30.3 ± 5.53 kg.m^-2^, p = 0.318), ASA score (3.00 ± 0.689 vs 3.00 ± 0.726, p = 0.727), and presence of cirrhosis (3 v 2, p = 0.868). The after-hours group had a significantly greater number of patients with prior abdominal procedures than the regular-hours group (15 vs 4, p = 0.0101) (Table 1).

**Table 1:** Preoperative Demographic Variables Values listed as median ± standard deviation where applicable

Variable	After-Hours	Regular-Hours	Total| p-Value
Patients (n)	26	20	
Age (years)	60.6 ± 18.1	63.9 ± 15.1	0.144
Sex (Male/Female)	9/17	4/16	0.275
Body Mass Index (kg/m2)	31.1 ± 6.38	30.3 ± 5.53	0.318
American Society of Anesthesiology Score (1-6)	3.00 ± 0.689	3.00 ± 0.726	0.727
Patients with Prior Abdominal Procedures (n)	15	4	0.0101
Cirrhosis (n)	3	2	0.868

There were no statistically significant differences between the two groups with regard to preoperative WBC (9.45 ± 3.88 vs 9.35 ± 4.09 x 103.µL-1, p = 0.565), neutrophil percentage (78.5 ± 14.1 vs 78.5 ±11.3, p = 0.501), Cre (1.2 ± 0.9 vs 1.0 ± 0.8 mg.dL-1, p = 0.360), and glucose level (135 ± 98.9 vs 116 ± 57.7 mg.dL-1, p = 0.244). There was a statistically significantly greater preoperative ALT (37 ± 20 vs 147 ± 80.5 U.L-1, p = 0.0017), AST (29 ± 19 vs 111 ± 91.5 U.L-1, p = 0.0001), AP (94 ± 77 vs 179 ± 103 U.L-1, p = 0.0168), and TBili (0.6 ± 1.0 vs 1.1 ± 1.7 mg.dL-1, p = 0.0147) in the regular-hours group (Table 2).

**Table 2:** Preoperative Laboratory Variables Values listed as median ± standard deviation where applicable

Variable	After-Hours	Regular-Hours	Total| p-Value
Patients (n)	26	20	46
White Blood Cell Count (x 10^3^.µL^-1^)	9.45 ± 3.38	9.35 ± 4.09	0.565
Neutrophil Percentage (%)	78.5 ± 14.1	78.5 ± 11.3	0.501
Glucose (mg.dL^-1^)	135 ± 98.9	116 ± 57.7	0.244
Creatinine (mg.dL^-1^)	1.2 ± 0.9	1.0 ± 0.8	0.360
Alanine Transaminase (IU.dL^-1^)	37 ± 20	147 ± 80.5	0.0017
Aspartate Transaminase (IU.dL^-1^)	29 ± 19	111 ± 91.5	0.0001
Alkaline Phosphatase (IU.dL^-1^)	94 ± 77	179 ± 103	0.0168
Total Bilirubin (mg.dL^-1^)	0.6 ± 1.0	1.1 ± 1.7	0.0147

On logistic regression analysis, the variables statistically significantly associated with the regular-hours group were preoperative AST and preoperative WBC, while a history of prior abdominal operations was associated with the after-hours group (Table 3).

**Table 3:** Logistic Regression Variables Associated With After-Hours Cholecystectomy Method = Stepwise, keep p < 0.05, remove p > 0.1

Variables Retained in the Model	Coefficient	Std Error	Wald	p-Value
Aspartate Transaminase	-0.0219	0.00994	7.13	0.0278
White Blood Cell Count	-0.376	0.181	4.33	0.0374
Prior Abdominal Surgery	3.73	1.50	5.63	0.0132

There were no statistically significant differences between the two groups with regard to any of the outcome variables of interest, including operative time (79.0 ± 28.9 vs 67.0 ± 23.9 mins, p = 0.196), EBL (25.0 ± 96.7 vs 25.0 ± 65.6 ml, p = 0.884), IVF given (917 ± 307 vs 800 ± 244 mL, p = 0.125), LOS (2.00 ± 1.12 vs 2.50 ± 1.76 days, p = 0.159), complications (0 ± 0 vs 0 ± 0, p = 0.849), 30-day mortality (0 vs 0), and 30-day readmission (1 vs 0). All patients attended follow-up in the surgery clinic within 30 days (Table 4).

**Table 4:** Postoperative Variables Values listed as median ± standard deviation where applicable

Variable	After-Hours	Regular-Hours	Total| p-Value
Patients (n)	26	20	46
Operative Time (min)	79.0 ± 28.9	67.0 ± 23.9	0.196
Estimated Blood Loss (ml)	25.0 ± 96.7	25.0 ± 65.6	0.884
Intravenous Fluids (IVF) Given (ml)	917 ± 307	800 ± 244	0.125
Conversion to ‘open’	0	0	N/A
Length of Stay (days)	2.0 ± 1.12	2.5 ± 1.76	0.159
Complications	1 (II)	1 (II)	0.849
30-day Readmission	1/26	0/20	N/A
30-day Mortality	0	0	N/A

The complications were as follows: one patient with cirrhosis in the regular-hours group required a single unit of fresh frozen plasma during surgery. One patient with cirrhosis in the after-hours group required a single unit of platelet transfusion. One readmission in the after-hours group occurred; this was due to a peri-stomal issue from the patient’s preoperative ostomy unrelated to the index cholecystectomy. There were no bile duct injuries.

On pathologic analysis, there was no statistically significant difference between the two groups with regard to the percentage rate of acute cholecystitis (69% vs 50%, p = 0.280). Despite all patients meeting the 2018 Tokyo criteria for acute cholecystitis, only 28 of 46 (60.9%) patients had acute cholecystitis noted by the pathologist, an additional 16 patients had chronic cholecystitis and 2 patients were noted to have neither. All patients had stones or sludge identified on the final pathology (Table 5).

**Table 5:** Pathology Pathologic findings in patients who underwent "after-hours" and "regular-hours" robotic cholecystectomy.

Pathologic Finding	After hours	Regular hours	p-value
Acute Cholecystitis	18/26	10/20	0.185
Chronic Cholecystitis	25/26	19/20	0.849
Necrotizing Cholecystitis	3/26	1/20	0.435
Gallbladder Empyema	5/26	3/20	0.141
Cholelithiasis or Sludge	26/26	20/20	1.00

## Discussion

There is a paucity of literature demonstrating the feasibility, safety, and efficacy of robotic surgery during the weekends and evenings. The aim of our paper, therefore, is to add to the literature addressing this topic, in particular by demonstrating the safety and feasibility of robotic cholecystectomy during hours when sub-optimal operating room staffing may occur.

In a 2023 review article by the World Society of Emergency Surgery, the authors noted that access is very limited for surgeons performing emergency procedures in general, let alone weekends and evenings [8]. Sudan et al. published two cases of robotic surgery to rescue patients from complications of bariatric surgery, a gastrointestinal leak at midnight, and a stricturoplasty on the weekend [9]. The authors are to be lauded for their ground-breaking publication, but the paper is now over 10 years old, and substantially more cases need to be studied. Of great interest is a case series of 10 emergency robotic colorectal operations successfully completed by Maertans et al. during the COVID-19 pandemic [10]. Unfortunately, their paper did not address the issue of after-hours access. In a study comparing 24 robotic and 20 laparoscopic marginal ulcer repairs, the authors Robinson et al. noted that a majority of their robotic operations occurred either in the evening or the weekend [11]. Although they did not perform a comparison against procedures done during regular hours, the robotic cohort as a whole did well with few complications.

Our paper is the only case-control study analyzing the outcomes between after-hours and regular-hours robotic cholecystectomy; its demonstration of equity in outcome will help guide hospitals and doctors in the burgeoning field of robotic surgery emerging from one of the greatest pandemics of the 21st century. Just as constant ERCP access can improve outcomes in cholangitis, constant access to superior minimally invasive technology should be considered as the next step in advancing care for patients with cholecystitis [12].

Although the robotic platform can improve certain general surgery outcomes, it requires staff accustomed to the technology [13]. An upfront investment via purchase of the platform, retaining trained staff, and replacement of expired instruments adds to the cost [14]. Even with such a system in place, just compensation must be offered to nurses and scrub technicians to provide coverage during weekends and evenings, rather than unfairly burden a Locum nurse or technician with a complex system they may not be familiar with. Given these hurdles, most (57%) general surgeons are denied this technology after regular staffing hours as a convenient way to brush aside this challenge to meet more difficult surgical diseases [15]. This is unfortunate given that patients with severe pathology, who often do not or cannot present to their primary care doctor for a timely outpatient referral, may instead present on the weekends and evenings because they cannot afford to miss their regular hours of employment [16,17]. Thus, the technology is restricted to the point where its benefit may be greatest.

This study shows that robotic cholecystectomy, one of the most commonly performed operations by a general surgeon on-call, may provide equally efficacious and safe outcomes during evenings and weekends (what we defined in this study as “after-hours”) as it does during “regular hours”. As the patient population ages and develops greater levels of insulin resistance and obesity, arming surgeons with robotic technology to treat diseases, that have become increasingly more severe, at any time of the day, will allow doctors to meet greater challenges with superior technology [18].

One of the strengths of this study is that it has the greatest number of after-hours robot emergency operations from a single surgeon. The study also demonstrated similar operating times between the two groups, which is likely one of the key factors that discourages the use of the robotic platform after-hours - the fear of a prolonged robotic operation without adequate support. Operative time increases cost. By counseling and constantly guiding staff, it is possible that nurses and technicians with limited but some familiarity with robotic surgery can be patiently guided by an experienced robotic surgeon to facilitate a smooth operation, as often occurred at the studied institution. Equivalent outcomes were obtained despite the after-hours group having more prior abdominal procedures, which can potentially increase operative time with lysis of adhesions.

Utilizing one surgeon at one hospital eliminates differences in technical skill and institutional practices as confounders, though it may reduce reproducibility to other hospitals. Nonetheless, similar to the studied institution, many hospitals across the country are suffering from high staff turnover with limited long-term technical experience available in the operating room outside of the practicing surgeon. It is in these hospitals that this paper will provide the most guidance.

In addition, good outcomes were attained in terms of bleeding, IVF given, length of stay, and readmission rates. There were no bile duct injuries in either group. IVF given during surgery was studied as it was hypothesized to be a good hallmark of operative complexity - a more challenging abdominal pathologic process may result in perioperative hypovolemia either by bleeding, poor PO intake prior to presentation, or inflammation resulting in fluid sequestration.

The statistically significantly greater liver function test derangements noted in the regular-hours group should not dissuade from the final result, since the clinical significance of the actual difference does not appear to be great. For instance, while the total bilirubin in the regular hours group averaged 1.1, this value is still considered within normal limits. On multivariate logistic regression, only AST was significantly associated with regular-hours cholecystectomy. Conversely, a greater number of patients undergoing after-hours robotic cholecystectomy had acute cholecystitis noted on final pathology (69% vs 50%); however, this was not a significant difference (p = 0.185).

The weaknesses of this study are its retrospective nature and small number of cases. Prospective studies with a robust number of cases at this time cannot answer the question of after-hours robotic cholecystectomy because few surgeons plan to perform surgery during these hours, and access is restricted. Small studies such as this one can broaden the question of why access should be restricted, encouraging more cases to be reported or pooled for further studies, including prospective ones.

## Conclusions

Cholecystectomy remains one of the most commonly performed operations by on-call general surgeons in the United States. Performing it safely remains paramount in regular discussions of this operation. Part of that discussion must expand into utilizing superior technology with availability after regular hours. While this paper has a limited number of cases, it should question restriction of access to the robotic platform after regular staffing hours. In turn, other surgeons may request to carefully utilize this important technology in situations where their experience can provide leadership to less knowledgeable staff. Hospitals should consider mobilizing the resources necessary to establish 24-hour robotic platform access to genuinely fulfill their duties as 24 hour-per-day providers of complex care.
